# Do weight management programmes delivered at professional football clubs attract and engage high risk men? A mixed-methods study

**DOI:** 10.1186/1471-2458-14-50

**Published:** 2014-01-21

**Authors:** Kate Hunt, Cindy M Gray, Alice Maclean, Susan Smillie, Christopher Bunn, Sally Wyke

**Affiliations:** 1 MRC/CSO Social and Public Health Sciences Unit, University of Glasgow, 4 Lilybank Gardens, Glasgow G12 8RZ, UK; 2 Institute of Health and Wellbeing, University of Glasgow, 27 Bute Gardens, Glasgow G12 8RS, UK

**Keywords:** Obesity, Weight management, Gender, Men, Masculinity, Evaluation, Randomised controlled trial, Qualitative

## Abstract

**Background:**

The prevalence of obesity in men in the UK is amongst the highest in Europe but men are less likely than women to use existing weight loss programmes. Developing weight management programmes which are appealing and acceptable to men is a public health priority. Football Fans in Training (FFIT), a men-only weight management programme delivered to groups of men at top professional football clubs, encourages men to lose weight by working with, not against, cultural ideals of masculinity. To inform further development of interventions in football club settings, the current study explored *who* is attracted to FFIT and *why* overweight/obese men choose to take part.

**Methods:**

A mixed-methods study analysing baseline data on 747 men aged 35–65 years with BMI ≥ 28 kg/m^2^ who were participants in a randomised controlled trial of FFIT, and data from 13 focus group discussions with 63 men who had attended the programme.

**Results:**

Objectively-measured mean body mass index was 35.3 kg/m^2^ (sd 4.9). Overall over 90% of participants were at very high or extremely high risk of future ill-health. Around three-quarters of participants in all age groups were at ‘very high’ risk of type 2 diabetes, hypertension and cardiovascular disease (72%, 73% and 80% of men aged 35–44, 45–54 and 55–64 years respectively). A further 21%, 16% and 13% were at ‘extremely high’ risk. Qualitative data revealed that the powerful ‘draw’ of the football club attracted men otherwise reluctant to attend existing weight management programmes. The location and style of delivery of early FFIT sessions fostered team spirit; men appreciated being with others ‘like them’ and the opportunity to undertake weight management in circumstances that enhanced physical and symbolic proximity to something they valued highly, the football club.

**Conclusions:**

The delivery of a weight management intervention via professional football clubs attracted men at high risk of ill-health. The setting enabled men to join a weight management programme in circumstances that felt ‘right’ rather than threatening to themselves as men. FFIT is an example of how to facilitate health promotion activities in a way that is consistent with, rather than challenging to, common ideals of masculinity.

## Background

Obesity has ‘escalated’ in recent decades [1] and the prevalence in UK men is amongst the highest in Europe [2]. More men are overweight or obese (body mass index (BMI) ≥ 25 kg/m^2^, 67%) than women (57%). In mid to late working life (35–64 years), more than three quarters of men are overweight or obese, and a third are obese, with the prevalence of obesity increasing with age (BMI ≥ 30 kg/m^2^ at: 35–44 years = 29%; 45–54 years =32%; 55–64 years = 35%) [3]. On current trends, nearly 50% of UK men will be obese by 2030 [4]; these trends pose a threat to individual health (through increased risk of cardiovascular disease, diabetes and cancer), a burden on health services and a challenge to public health [1,4-7].

A 5-10% weight loss can produce health benefits [8], but there are real challenges in encouraging weight loss in men. Not only is obesity rising faster in men than women, but men seldom use existing commercial and National Health Service (NHS) weight loss programmes [9-12]. Furthermore, many men seek a body weight that is not consistent with biomedical definitions of ‘normal weight’, ‘overweight’ and ‘obesity’ [13-15] and are more willing to challenge these definitions than women [16]. Men often view dieting as ‘feminine’ [17,18], are more likely to use exercise to control their weight [19], are more resistant to healthy-eating campaigns [20], and are less aware of links between diet and ill-health [19,21]. Thus, as argued elsewhere [22], prevailing cultural constructions of masculinities are closely related to men’s decisions about health-related behaviours, including help-seeking [23-25] and alcohol consumption [26-28], as well as eating, physical activity (PA) and weight loss. These constructions are often said to be inherently health-damaging [29], mitigating against men taking care of their health, or putting them in an ambiguous position between wanting to care for their health as responsible citizens, without appearing to care ‘too much’ as men [30]. Thus, with the increasingly male hue of the obesity epidemic, developing weight management strategies which are appealing and acceptable to men is a public health priority [31], and most likely to be successful when working with, not against, cultural ideals of masculinity. In other words, health-promoting interventions need to engage men without being an anathema to valued aspects of their identities [22]. Other weight management programmes for men take a similar view and have shown promising results [31-33], although evidence on what works in weight loss for men is relatively sparse [34] given their underrepresentation in weight loss trials [35].

In order to inform further development of weight management and other public health interventions in football club settings this paper reports firstly, on the characteristics of 747 men participating in a randomised controlled trial (RCT, ISRCTN32677491) of a weight management programme [36] designed specifically to attract men at high risk of ill-health due to obesity [37], and, secondly, their accounts (as expressed in 13 focus groups with 63 men) of why they decided to participate in the programme. Football Fans in Training (FFIT) is a men-only, evidence-based [8,38], 12-session, weight management and physical activity group programme with subsequent minimal-contact weight loss maintenance support delivered free of charge at Scotland’s top professional football clubs by community coaches trained in diet, nutrition, physical activity and behaviour change techniques to a standard programme delivery protocol [37]. This training included discussion of SMART goal setting and implementing the pedometer-based walking programme. Training was interactive and designed to promote the principles of adult learning and the use of banter in the group sessions to make men feel more comfortable about raising sensitive issues. Coaches shared ideas about suitable in-stadia physical activities which could be adapted to different levels of fitness.

FFIT exploits the traditionally male environment of football clubs [39], existing loyalty to football teams and the opportunity to participate in men-only groups to maximise men’s engagement [32]; its development is described elsewhere [37]. In brief, FFIT is gender-sensitised in context, content and style through its delivery at professional football stadia by club community coaches who encourage participative learning, a practical focus through shared experiences of progression towards behaviour change and a light-hearted atmosphere (‘banter’) which supports men in discussing potentially difficult issues. In addition to advice on diet, alcohol and sustainable behaviour change strategies, FFIT focuses on physical activity through an incremental pedometer-based walking programme to encourage greater activity in daily life [22] and pitch-side sessions led by club coaches. FFIT’s location within professional football clubs reflects growing recognition of the potential of sporting organisations to deliver health initiatives to men [40,41]. Club community coaches receive two days training to ensure that key elements of FFIT are delivered. The mapping of these key elements onto behaviour change techniques which are effective in weight management [42] (e.g. self-monitoring of weight and physical activity, intention formation, goal setting and review) is described elsewhere [37]. The results of a feasibility study [43] and semi-structured telephone interviews with participants in the pilot deliveries of FFIT in 2010 [22] suggested that the programme had potential to help men to lose weight and is popular with participants. The results of a full-scale RCT, the first such study to be conducted in a professional sports setting, powered to assess whether participation in FFIT helps overweight/obese men to lose at least 5% of their body weight 12 months after baseline (pre-programme) measurement provides evidence of the effectiveness and cost effectiveness of FFIT [36].

## Methods

### Participants

The data were gathered as part of the FFIT RCT [36]. Within a 3.5 month period^a^ prior to start of the first delivery (August/September 2011) of the optimised FFIT programme in 13 clubs (12 in the Scottish Premier League and one recently relegated to a lower division), sufficient men were recruited to fill all available places (at that time funding from the Football Pools and Scottish Government was available for deliveries at the 13 clubs in August-December 2011, February-April 2012 and August-December 2012). Following measurement at baseline in August-September 2011 and assessment of eligibility (age 35–65 years, BMI > 28 kg/m^2^), 374 men were randomly allocated to undertake FFIT immediately (intervention group) and 374 to undertake FFIT 12 months later (waitlist comparison group). Men who were unable to attend baseline measures were placed on a waiting list and offered a place on the February 2012 delivery of FFIT (‘non-trial’ group) if any places remained after prior allocation to men who had attended the measurement sessions [36]. We report the characteristics of the 747 men who took part in the RCT, excluding one man randomised to the comparison group who subsequently withdrew.

### Ethics

Ethical approval was granted by the University of Glasgow College of Social Sciences Ethics Committee (CSS/2011/029), which complies with the UK Economic and Social Research Council’s Framework for Research Ethics. Participants gave separate written informed consent for participation in the RCT and the focus group discussions. Men were offered travel expenses and a £20 football club shop voucher as a gesture of thanks for their contribution to the focus group discussions.

### Measures

All objective baseline physical measures were taken in the clubs by fieldworkers trained to standard protocols [36]. Weight (kg) was recorded using an electronic scale (Tanita HD 352), with participants wearing light clothing, no shoes and having emptied their pockets. Height (m) was measured (without shoes) using a portable stadiometer (Seca Leicester). Waist circumference (cm) was obtained using a 200 cm tape measure. At least two measurements were taken, followed by a third if these differed by ≥5 mm; the mean of all measures was calculated. Resting blood pressure (BP) was measured using a digital BP monitor (Omron HEM-705CP). Equipment was calibrated prior to fieldwork.

Descriptive statistics for weight, BMI, blood pressure and waist were calculated. Categories of waist circumference (‘low’ < 94 cm; ‘high’ ≥ 94 cm and <102 cm; ‘very high’ ≥ 102 cm) and BMI (‘overweight’ BMI 28–29.99; ‘mild obesity’ BMI 30–34.99; ‘moderate obesity’ BMI 35–39.99 and ‘extreme obesity’ BMI >40) were used to assess risk of future ill-health [38] so we could compare the health risk of FFIT participants with similarly-aged men in the Scottish population [3].

### Focus groups participants, methods and analysis

As part of the process evaluation of FFIT [36], men who had attended at least six FFIT sessions in August-December 2011 (85% of intervention group) were invited to a discussion about their experiences of FFIT at the end of the 12 weekly FFIT sessions. Of 295 men contacted, 133 (45%) indicated willingness to take part. At each club, up to 6 of these men, chosen randomly, were invited to a 60-minute discussion at the club stadium; 63 men participated in 13 focus groups. These were audio- and video-recorded with consent, transcribed verbatim, and transcripts checked for accuracy against recordings. To thank them for their time, men were offered a £20 club shop voucher and travel expenses. During the discussions men were asked about several aspects of FFIT, including why they signed up in the first place and early experiences of attending FFIT, as reported here.

The focus group data were analysed using a structured, thematic approach [44]. Transcripts were read by several authors to identify broad themes, guided in part by our research questions in the process evaluation. Transcripts were then coded to broad themes including the ‘draw’ of the football club/club setting, reasons for participating, and satisfaction with and acceptability of FFIT; coding was quality-checked by another researcher.

Secondly, the content of the themes relevant to this paper was examined in detail by a third researcher. Each coded extract was read line by line to identify all sub-themes. These sub-themes were summarised schematically and each occurrence of every sub-theme was noted and compared with data from subsequent groups, using the OSOP method [44], which allows systematic comparison of data from different participants/groups, noting anticipated and unanticipated themes [45]. Attention was paid to ‘deviant cases’ to ensure all perspectives were captured [45]. We paid particular attention to a code entitled ‘negatives of club setting’; this contained relatively little material (from just 2/13 clubs) and can be summarised as disappointment that the more ‘professional’ side of the club did not show the same interest in FFIT participants that the community coaches had done.

## Results

We first describe the characteristics of men who enrolled for FFIT and their risk of future disease (on the basis of their weight and body compositional measures) in comparison with Scottish men of the same age. We then present qualitative data to show what attracted men to the FFIT programme, focusing both on ‘push’ factors (such as growing health concerns, wanting to lose weight to ‘be there’ for their family in the future) and ‘pull’ factors, specifically the attraction or ‘draw’ of the football club setting.

### Characteristics of men attracted to FFIT

The baseline characteristics show that FFIT succeeded in attracting men at high risk of future ill health (Table 1); mean weight was 109.5 kg (sd 17.3) and mean BMI was 35.3 kg/m^2^ (sd 4.9). Over 90% of participants had a BMI ≥ 30; 44% were classed as ‘mildly obese’, 31% as ‘moderately obese’, and 17% as ‘extremely obese’. Mean age was 47.1 years (sd 8.0), mean waist circumference (WC) was 118.4 cm (sd 11.7); all but 2 men had a ‘high’ (4%) or ‘very high’ WC (96%). Mean systolic and diastolic blood pressure readings were 140 and 89 mmHg, respectively.

**Table 1:** Baseline characteristics of men recruited to FFIT RCT (n = 747)

	**mean (sd) or n (%)**
**Mean age (sd) (years)**	47.1 (7.98)
**Mean weight (sd) (kg)**	109.5 (17.26)
**Mean BMI (sd) (kg/m**^**2**^**)**	35.3 (4.91)
**BMI category (n,%)**	
Overweight (BMI 28–29.99)	57 (7.6)
Obese I - ‘Mild’ obesity (BMI 30–34.99)	329 (44.0)
Obese II - ‘Moderate’ obesity (BMI 35–39.99)	233 (31.2)
Obese III - ‘Extreme’ obesity (BMI ≥40)	128 (17.1)
**Mean systolic blood pressure (sd) (mmHg)**	140.3 (16.31)
**Mean diastolic blood pressure (sd) (mmHg)**	88.8 (10.21)
**Mean waist (sd) (cms)**	118.4 (11.74)
**Waist circumference category (n,%)**	
<94 cm (‘low’)	2 (0.3)
94–102 cm (‘high’)	30 (4.0)
>102 cm (‘very high’)	715 (95.7)
**Weight loss activities in previous 3 months (n,%)**	
*Attended a commercial weight loss programme*	
Not at all	718 (96.4)
At least 1–2 times per month	27 (3.6)
*Missing*	*2*
*Attended a weight reduction clinic at your GP surgery or another NHS setting*	
Not at all	731 (98.3)
At least 1–2 times per month	13 (1.7)
*Missing*	*3*

Table 2 compares FFIT RCT participants with male Scottish Health Survey respondents of similar ages [3] according to health risk category. FFIT participants were at much higher risk of future ill-health (type-2 diabetes, hypertension and cardiovascular disease); around ten times more men were classed as being at ‘extremely high’ risk (21%, 16% and 12% of FFIT participants aged 35–44, 45–54 and 55–65 years, respectively, compared with 2%, 2% and 1% of Scottish men of the same age). A further 223/311 (72%) of FFIT participants aged 35–44, 212/290 (73%), aged 45–54, and 116/146 (80%) aged 55–65 years were at ‘very high’ risk, compared with 25%, 25% and 32% of Scottish men. Although participants were sufficiently concerned about their weight to enrol for FFIT, less than 4% had attended a commercial or NHS weight loss programme or clinic in the previous 3 months (Table 1).

**Table 2:** **Comparison of health risk category of men recruited to FFIT RCT with men in the Scottish general population by age group from the 2011 Scottish Heath Survey (SHeS)**^**+**^

**Waist circumference**	**Health risk category**^***c***^		**Age categories (years)**								
**(WC)**^***a ***^**and BMI classification**^***b***^		**All FFIT**	** FFIT 35-44**		***SHeS 35-44***	** FFIT 45-54**		***SHeS 45-54***	** FFIT 55-65**		***SHeS 55-64***
		**n**	**n**	**%**	***%***	**n**	**%**	***%***	**n**	**%**	***%***
**Overweight**											
Low WC	No increased risk	2	1	0.3	*19.4*	1	0.3	*14.6*	0	0.0	*9.3*
High WC	Increased	18	4	1.3	*17.7*	10	3.4	*19.7*	4	2.7	*21.3*
Very high WC	High	37	13	4.2	*6.2*	17	5.9	*8.2*	7	4.8	*16.4*
**All overweight**		**57**	**18**	**5.8**	***43.2***	**28**	**9.6**	***42.5***	**11**	**7.5**	***47.0***
**Obesity I**											
Low WC	Increased	0	0	1.9	*0.4*	0	0.0	*1.2*	0	0.0	*-*
High WC	High	11	6	0.0	*2.8*	3	1.0	*5.8*	2	1.4	*0.9*
Very high WC	Very high	318	114	36.7	*22.4*	131	45.2	*18.3*	73	50.0	*25.5*
**All obese I**		**329**	**120**	**38.6**	***25.7***	**134**	**46.2**	***25.3***	**75**	**51.4**	***26.5***
**Obesity II**											
Low WC	Very high	0	0	0.0	*-*	0	0.0	*-*	0	0.0	*-*
High WC	Very high	1	0	0.0	*-*	1	0.3	*-*	0	0.0	*-*
Very high WC	Very high	232	109	35.1	*2.8*	80	27.6	*6.3*	43	29.5	*6.9*
**All obese II**	**Very high**	**233**	**109**	**35.1**	***2.8***	**81**	**27.9**	***6.3***	**43**	**29.5**	**6.9**
**Obesity III**											
Low WC	Extremely high	0	0	0.0	*-*	0	0.0	*-*	0	0.0	*-*
High WC	Extremely high	0	0	0.0	*-*	0	0.0	*-*	0	0.0	*-*
Very high WC	Extremely high	128	64	20.6	*1.7*	47	16.2	*2.0*	17	11.6	*1.1*
**All obese III**	**Extremely high**	**128**	**64**	**20.6**	***1.7***	**47**	**16.2**	***2.0***	**17**	**11.6**	***1.1***
**Total**		**747**	**311**	**100.0**	*(73.4)*	**290**	**99.9**	*(76.1)*	**146**	**100.0**	*(81.5)*

+ Table adapted from Table 7.5, Scottish Health Survey (SHeS), 2011 (Volume 1 Adults [3]) to allow direct comparison with men of comparable ages in the Scottish population in 2008–2011.

***a*** Waist circumference categories according to WHO/SIGN guidance – **low**: <94 cm; **high**: ≥94 cm and <102 cm; **very high** ≥102 cm; ***b*** BMI categories as defined in Table 1; ***c*** Health risk category according to SIGN guidelines [37].

### What attracted men to FFIT?

Having shown that FFIT attracted high risk men who were not participating in alternative programmes, we consider what attracted them to FFIT. Analysis of the focus groups revealed a combination of ‘push’ and ‘pull’ factors were crucial in men’s initial attraction to FFIT.

**Figure 1:**
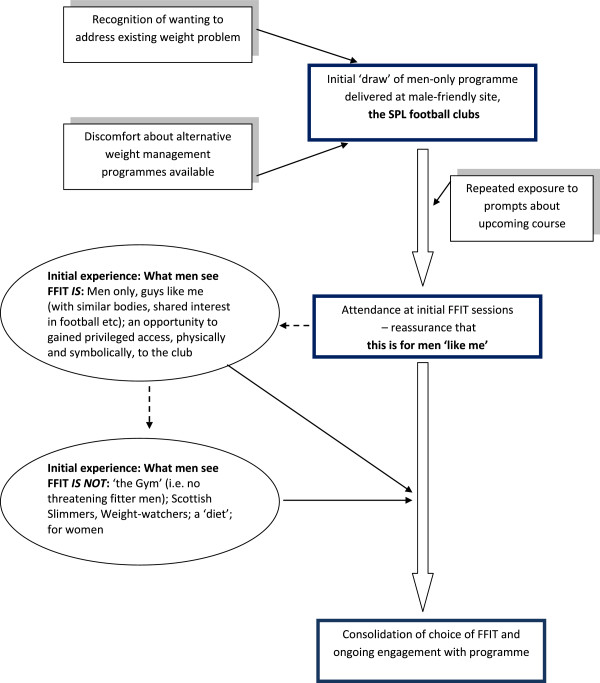
Factors attracting men to take part in FFIT – the power of the ‘draw’ of the football club for themselves and other like-minded/like-bodied men.

The most important factor was the opportunity to undertake the FFIT programme at the *football club* itself (see Figure 1). Many men articulated the combined importance of ‘knowing’ they ‘needed’ to do something about their weight/poor physical fitness, *and* the powerful draw of attending a weekly programme at the club (which they had often supported since boyhood):

**M1**: *I’ve struggled with my weight since, maybe, early-twenties and I’ve tried various diets, various things, and you seem to get to a stage where you’re successful, then you fall back out the way again. So, when I seen this advertised in the paper … the main thing that drew us to it was because it’s [Club07]. You’re going to be involved at [Club07], whether it just be at the ground, stadium … That was what really attracted me to it.* (Club07 FG)

**M2**: *I was very aware that, every time I was buying a new suit… the trouser size was getting bigger, and I just wasn’t happy with that, and I just wanted to address it. And with it being, having a tie in with the team I’ve supported all my life, I felt that the two kind of – they, it fitted nicely. It meant I could do something and I could maybe get a wee sneaky peek behind the scenes at [Club04 ground]*. (Club04 FG)

Whilst some said they wanted to do something about their weight for themselves, others linked concerns about their weight, health or fitness to family obligations and aspirations:

**M3:***I’ve had two kids in the last three years… that was part of the motivation, as well – just getting fitter for my kids… I thought, “I need to get myself fit for these weans* [children], *you know? I need to be aboot* [about] *for as long as possible.”* (Club12 FG)

Even with the combined *motivation* to do something about their weight (a ‘push’ factor) and the *opportunity* to do this in a place they valued (the draw of the club, a ‘pull’ factor), some men talked explicitly about the apprehension they felt about enrolling in a weight management programme prior to arriving at the club, underlining the barriers many felt about joining a weight management group (Extract 1, Table 3). Others expressed worries in different ways, such as a fear of being so overweight, unwell or unfit that they might be rejected if they applied for FFIT (Extract 2, Table 3). Indeed, many men indicated they heard about FFIT from several sources (e.g. when approached by fieldstaff at pre-season games, adverts or features in the media or workplace, word of mouth) before deciding to enrol.

**Table 3:** Examples of verbatim quotes illustrating how the football setting acted as a ‘draw’, overcoming obstacles and attracting men ‘like’ them

	**Verbatim quotes**
**The draw of the football club overcoming anxiety about enrolling in a weight management programme**	**Extract 1**
	M4: I remember the first time… when you got weighed and all that sort of stuff – if they were going to take you on the course – and I genuinely, I just didnae want to be here. I felt very nervous aboot the whole thing, and he [fieldstaff member] was getting the weight. It was very embarrassing. It’s not now, but at the time, it was. At the time, I was like, “Oh, this is terrible,” and aye, measuring my waist, and I came in here … That was the first time I met [Coach 1], and I genuinely thought, “After I get out [of] here, I’m just going in the car, ignore the phone calls, ignore the emails, I’m not daen [doing] that. That’s ridiculous.” And I sat in here with [Coach 1], and he was telling us, “Right, you know, of course we train here, at [Club07 ground].” And I thought, “That’s good. I wouldn’t mind training here at [Club07 ground]....” And after that, he said, “Aye, ken what we’ll do, we’ll get you some training gear … you’ll be running aboot in the training gear.” And honestly, I couldnae get the grin off my face. I was like a bairn [child]. I thought, “I want to be training at [Club07 ground],” with the, you know, aye, with the kit. (Club07 FG)
	**Extract 2**
	M4: I just heard, you know, seen something about it – I don’t know if it was ‘Good Morning Scotland’[BBC radio programme] or one of the radio programs. I’d heard something about it, but I never thought anything more of it, ‘til I … was approached [at a pre-season game] and signed up, and then I felt quite apprehensive. I thought, “Och, no,” you know? I’ve got arthritis, I’m taking these pills, I can hardly move, you know? So, you know, they’ll probably reject me, you know, when I go along”. So I thought, “Well, I’ve spent fifty years looking after other people, so it’s maybe time to look after myself” – but I came along and was very pleasantly surprised, and my goal was to lose weight, coz I was over fourteen stone, and to get fit. (Club02 FG)
**Attending FFIT enrolment and early sessions and seeing that this is for people (men) ‘like me’**	**Extract 3**
	M1: Aye, that was the thing, it was… Everybody was starting from the same spot.
	M3: Starting point.
	M1: And it wasnae super fit people and going to completely the other scale – we were all kinda in the same spot, to start off wi’. That was why I did it. (Club13 FG)
	**Extract 4**
	M1: The good thing was, straight from the start, we all had something in common with each other. Rather than being sixteen strangers, we’d all something in common, and that was the club and a love for it.
	M5: Two things in common. We were fat and we supported [Club03].
	M1: Sorry, we were cuddly and supported [Club03], and that was the big factor. So no matter… you didn’t know each other’s names, we immediately were able to converse with each other easily. (Club03 FG)

After the difficult process of deciding to at least attend the baseline measurement session, many men expressed relief during the initial stages to see they were amongst men ‘like them’ in crucial ways. They recognised themselves in the physiques and perceived levels of fitness of other men there, and they were able to take for granted a shared interest in football and the club (Extract 3, Table 3). Often their remarks about this recognition reflected the self-deprecating humour and ‘banter’ which characterised FFIT’s style of the delivery [37] (Extract 4, Table 3).

This initial reassurance they had chosen the ‘right’ programme for them was quickly reinforced by experiences of key aspects of the first few sessions which consolidated the feeling that they were undertaking a programme somewhere where they wanted to be (Figure 1). Men articulated this in relation to gaining privileged access to club facilities (e.g. home/away changing rooms, the tunnel, pitch side):

**M1**: *I’m a fan, and I think that helped a lot… the fact that I was coming to [Club05] stadium and going into the changing room and stuff like that… I mean, it was just nice. And you felt kinda part of it…*

**M3**: *The fact, walking about the stadium, up and doon* [down] *the stairs.* (Club05 FG)

**M1**: *Just the enjoyment of coming along and being involved in the club… even walking round [Club04 stadium], to me, was an exciting part of the Monday night… walking up and down the terraces. You might only be restricted to one area when you come to a game, but, you know, the fact that you’ve got carte blanche, you can go wherever you like, other than the pitch*. (Club04 FG)

Participants also talked enthusiastically about gaining an ‘insider’ view of the club through their contact with the community coaches (supplemented at some clubs by visits from club celebrities, such as (ex-)players). This ‘insider’ view was reinforced by a shared commitment to the club and their informal banter with like-minded men, both coaches and fellow participants:

**M5**: *These guys* [FFIT coaches]*… have got an insight to the club. These guys are [Club03], through and through. They are. They’re us. They’re the same as us… It’s in the blood.*

**M6**: *Even just daft things like asking… them things like, “What’s happening aboot* [about] *the football? What’s happening?”*…

**M3**: *Aye, you get good insight into it.*

**M6**: *Wee bits of banter, round, and stuff that nobody else could have told you.... you just felt as if you were interacting with guys that were a part of [Club03].*

**M4**: *You feel you’re going into the team, a wee bit, don’t you?* (Club03 FG)

As illustrated here, this ‘insider’ experience gave participants a sense of physical *and* symbolic proximity to the club. This rapidly fostered a group identity, a sense of being part of a mutually supportive team despite a diversity of backgrounds and socioeconomic circumstances:

**M5**: *We were all from different backgrounds, but everybody bonds, you know? And we’ve went fae* [from] *the milkman to the postman to the office worker to the sales guy, but everybody just bonded and it didnae matter whether you were from Falkirk or from Glasgow.*

**M2**: *Aye, and everybody, when it came to the team building, that was what got me. Everybody gelled really quick and there was nae heroes in it. Everybody helped each other out, whether they were struggling with whatever exercise it was – everybody kinda pulled thegether* [together]*, and that helped you through it. Whereas, in a gym, you don’t get that. The guys just laugh at you, just think, “You’re wasting your time on that machine, I could be on it.”…*

**M1**: *There was a team spirit and you didnae want to let the team down* (Club03 FG)

Thus, their choice to sign up for FFIT seemed all the more ‘right’ given their instant recognition that the programme attracted other men ‘like them’, and their early experiences, which enhanced the physical and symbolic proximity to the football club that FFIT offered and the opportunity to be part of a mutually supportive ‘team’. One group articulated how this went beyond a simple sense of team spirit, to the heart of their identity, giving them a sense of *gaining* something highly valued through their participation:

**M5**: *There’s an ownership wi’ the club, or I suppose ‘ownership’ is probably not the right word, but you definitely feel a connection to the club that you maybe didn’t have before. Even just the t-shirts we all got.*

**M1**: *Aye, it’s like an identity. It’s almost as if… you become, I know this sounds crazy, but I had this in my head that it’s all like, you know, I’m part of the team, the fans’ team, you know?… I think that was one of the things that kept me going, as well*. (Club12 FG)

The feeling that the programme was ‘right’ for them was further reinforced by what men said that FFIT was *not* (Table 4). In emphasising that FFIT was *not* for women, *not* Weight Watchers or Scottish Slimmers, *not* a diet club, and so on, the men underlined how FFIT enabled them to take on what might otherwise be seen by themselves (or others) as a feminised activity (deliberate attempts to lose weight) and reclaim it as something which enhanced their sense of themselves as men.

**Table 4:** Examples of verbatim quotes illustrating what FFIT is NOT

	**Verbatim quotes**
**FFIT is *NOT*: a ‘diet club’, the gym, for women**	**Extract 1**
	**M5**: I think we were quite happy it was just men, to be honest. We spoke about going to Weight Watchers or that… but we were saying that, a lot of things are set up and it is mainly female – so it was good to see something that was just for male…
	**M1**: For guys.
	**M2**: I’m not being sexist or anything, but…
	**M1**: That’s right. I think the majority felt that Weight Watchers was not for us. That was very much a women’s thing.
	**M2**: That’s a ladies’ thing.
	**M1**: And, sorry for being sexist, here, but it’s, I think the majority felt no, Weight Watchers or Scottish Slimmers or whatever.
	**M5**: They’re always targeted at women.
	**M1**: Was targeted for women. It was never targeted for men… And even a lot of the exercise classes that gyms run are targeted for women and not for men.
	**M3**: I think ladies, to come to something that we’ve been on for the twelve weeks, wouldn’t maybe appreciate the language that’s used. You know, it’s all guys that’s there and, you know, it’s football.
	**M2**: Yeah, you see ‘cos, it was not exactly industrial language, but there was the odd bit of comment and football slagging and stuff like that. And yeah, we would laugh it off but somebody [?a woman] might not.
	**M3**: They might take offence to it.
	**M2**: They might do… I thought it was easier to build up the camaraderie without ladies being there. (Club08 FG)
	**Extract 2**
	**M4**: No, but blokes don’t diet, and I think if – blokes are like a wolf pack. If they get on together, they support each other really, really well, and if they don’t get on with each other, one goes off and he’ll go to his own devices, and if someone turned round and said to me this was a diet club, I would have said, “No, I’m not interested. Not interested.”
	**M1**: Yeah, that’s right.
	**M4**: But as it’s a way of life, and the way you’re looking at that and healthy eating and so on and so forth, I think when you do come here, you build up that camaraderie and you build up that friendship, and if you see each other, you say, “Hi, how’s it going?” If you wanna get in contact with certain people, you’ve got their email addresses and stuff, how’s it going – but I think if someone said diet, I don’t think any of us would have been here, to be fair. (Club06 FG)
	**Extract 3**
	**M1**: I mean, the common denominator is football. We all like football, you know? And that gets us all going.
	**M4**: Of course it does.
	**M3**: That was it.
	**M2**: The good thing, to me, is I didnae like going to a gym. I would prefer to go to a class, or have somebody telling you what to dae [do] and what not to dae. It was kinda like, I don’t want to go to a gym and see all the younger ones, the fitter ones – and you got a kinda, you look roon [around] and you think they’re looking at ye [you]. “He’s that fat he cannae [can’t] even dae that.” And that was the kinda thing I liked. You come here, everybody was in the same boat – everybody was between a certain age and overweight, ‘cos that’s how I got into it. (Club10 FG)
	**Extract 4**
	M1: Well, I would never go to a gym, to be honest. I’ve tried. Kept saying I was gonna do it, but I would have mucked about on my own for a while and I still wouldnae have done anything. But the fact it was [Club05] Football Club you were going to made it more palatable, you know?
	M3: Doesnae matter what club, ken, and if you’re into that club, I think you’d go, and it would spur you on. Rather than Weight Watchers.
	M1: The fact we got a top, as well, I think, you know? You had a training kit and the training top on and things like that, you were always quite.
	M3: The fact, walking about the stadium, up and doon the stairs. (Club05 FG)
	**Extract 5**
	M1: From day one, it was stressed in here, this is no a diet, it’s not a diet.
	M2: Yes, aye, uh huh. It’s not, it’s a change in lifestyle as much as anything. (Club01 FG)

## Discussion

With male levels of obesity rising, and men’s lower participation in existing weight management programmes [35], the need for innovation in the design and delivery of programmes to attract overweight/obese men has been recognised [31,33]. Professional sports clubs, and specifically professional football clubs in the UK, are increasingly seen as settings that can attract men to health promoting activities [41,46]; Pringle and colleagues suggest that “the product (i.e. football/EPL [English Premier League])… the place (club stadia and facilities), people (players and management) and processes (including communication, marketing and the product delivery infrastructure)” all contribute to their appeal [41]. If this ‘draw’ is to be used to maximum public health benefit, it is important that programmes delivered in these popular locations are based on best-evidence about what works, *and* reach those men most likely to benefit. Behavioural ‘choices’ are embedded in socio-cultural contexts, including those relating to gender, yet gender is often ignored in intervention development, perhaps explaining underuse by men (only 10-30% of participants in weight loss programmes are men [9-12]). This paper shows that FFIT, a gender sensitised, weight management programme delivered to standardised protocols across all participating clubs [37], has succeeded in attracting men whose weight and body composition puts them at very high risk of ill-health, and thus adds further evidence of the potential of the professional football club setting for attracting men to health-related initiatives, including weight management.

In explaining why FFIT succeeded in attracting them, men provided multiple and overlapping explanations for enrolling. These included ‘push’ factors in their personal and family lives and experiences, such as growing health concerns, wanting to lose weight to ‘be there’ for family, or to regain sufficient fitness to restart valued activities; and ‘pull’ factors, in this case specifically the ‘draw’ of the delivery of FFIT at football stadia. The COM-B framework for understanding behaviour change [47] suggests that people require *capability* (physical and psychological capacity to engage in behaviour, including skills), *opportunity* (external factors, including the physical environment and ‘social opportunity afforded by the cultural milieu’), and *motivation* (habitual processes, emotional responses, goals and conscious decision-making) to undertake behavioural change. We would argue that the professional football club setting appears to have provided these men with a physical and social opportunity which matches well with their own identities and values as men. The fact that FFIT attracted like-minded men with similar physiques and levels of fitness (‘people like them’) contributed to the appeal of the group format. In relation to the COM-B model, men’s accounts suggested they *wanted* to, and were *motivated* to, lose weight for whatever reason and that the FFIT programme was an ideal *opportunity*. Our attempts to ‘gender sensitise’ FFIT in context (professional football stadia), content (e.g. specific sessions on alcohol and weight, ‘branding’ with club insignia on programmes and club-based t-shirts) and style of delivery (participative, peer-supported, learning which encouraged male ‘banter’) appears to have worked in terms of engagement; the results of the RCT demonstrate the effectiveness of FFIT in achieving clinically significant weight loss at 12 months [36].

Assertions that men undertake ‘risky’ behaviours (such as smoking and drinking excessively) and avoid health-protective behaviours (such as help-seeking) to be ‘real’ men are commonplace [29], and constantly reinforced in popular media and day-to-day interactions. Vandello and Bosson argue that “people view the very state of manhood as a precarious social status that is hard won and easily lost, [requiring] continual public demonstrations of proof” (p101) [48]. This resonates with other conceptualisations about how people demonstrate in their day-to-day social interactions that they are behaving in ways that are appropriate for their gender [49]. De Visser and colleagues, for example, argue that taking part in activities which are deemed to be ‘feminine’ is one way in which men can ‘lose’ man points [50] or ‘masculine capital’ [51] (in which ‘masculinity’ is seen as a resource accumulated through social interaction that can be enhanced, undermined or destroyed by certain actions and behaviours). Because dieting and taking part in commercial weight loss programmes are so often constructed as ‘feminine’ activities [17,18,20,31,32,52], it is perhaps no surprise that men are more willing to engage in programmes that give them the easy opportunities to revalidate their credentials as men. This paper reinforces earlier evidence[22] that FFIT is valued by men who want to lose weight, not just because it is enjoyable and engaging, but because it enables men to ‘bolster’ their masculine capital through their association with football clubs, symbolically and physically, and their participation and association with other men like them.

### Strengths and limitations

The study has a number of strengths. Weight, height, blood pressure and waist circumference were objectively measured by fieldstaff trained to standard protocols who were independent of the clubs delivering FFIT, as were the researchers who facilitated the focus groups. This illustrates that whilst others have acknowledged the challenges of evaluative research in these contexts [53], it is possible to obtain objective measures and use evaluative designs (the ‘gold standard’ RCT) that normally reside more comfortably in clinical settings. The study also has limitations. Focus group participants were men who had completed the majority of FFIT sessions, and were willing to volunteer to make an additional trip to the club. It is thus possible their views are more positive than other participants. On the other hand, the location of the discussions may have been seen as a positive opportunity to meet fellow participants in a venue they enjoyed coming to. Certainly, the views expressed are consistent with those gathered via shorter telephone interviews with men who undertook the pilot deliveries of FFIT in August-September 2010 [37], with comments reported back by fieldworkers at the follow-up measurement sessions, and with feedback from the coaches.

## Conclusions

This mixed-methods study has shown that the delivery of a weight management intervention via professional football clubs attracted men who were at very high risk of ill-health, the majority of whom did not attend other weight management programmes. The familiar and valued football club setting, the opportunity to interact with others ‘like them’, and the gender-sensitised content combined to reassure men they were in the right place and to support their engagement with the programme. Football Fans in Training provides an excellent example of how to facilitate weight management in a way that is consistent with, rather than challenging to, common ideals of masculinity.

## Endnote

^a^Formal recruitment began on 2 June 2011 when funding for the RCT was confirmed. Men who had already enquired about the study in response to publicity about the pilot deliveries of FFIT were contacted once formal recruitment began.

## Competing interests

The authors declare that they have no competing interests.

## Authors’ contributions

KH, CMG and SW contributed to the research design. AMc and SS led data collection at the club stadia, with support from KH, CMG, and SW. KH and CMG performed the frequency analyses. KH, CMG, CB and SW read transcripts. KH performed the main qualitative analysis presented in this paper with additional input from CB and SW. KH drafted the manuscript. All authors commented on drafts, and read and approved the final manuscript.

## Pre-publication history

The pre-publication history for this paper can be accessed here:

http://www.biomedcentral.com/1471-2458/14/50/prepub
